# Optimal use of tocilizumab for severe and critical COVID-19: a systematic review and meta-analysis

**DOI:** 10.12688/f1000research.45046.1

**Published:** 2021-02-04

**Authors:** Cahyo Wibisono Nugroho, Satriyo Dwi Suryantoro, Yuliasih Yuliasih, Alfian Nur Rosyid, Tri Pudy Asmarawati, Lucky Andrianto, Herley Windo Setiawan, Bagus Aulia Mahdi, Choirina Windradi, Esthiningrum Dewi Agustin, Jonny Karunia Fajar

**Affiliations:** 1 Department of Internal Medicine, Faculty of Medicine, Airlangga University, Surabaya, East Java, 60132, Indonesia; 2 Universitas Airlangga Hospital, Surabaya, East Java, 60115, Indonesia; 3 Department of Pulmonology and Respiratory Medicine, Faculty of Medicine, Airlangga University, Surabaya, East Java, 60132, Indonesia; 4 Department of Anesthesiology and Reanimation, Faculty of Medicine, Airlangga University, Surabaya, East Java, 60132, Indonesia; 5 Faculty of Medicine, Airlangga University, Surabaya, East Java, 60132, Indonesia; 6 Department of Internal Medicine, Faculty of Medicine, Brawijaya University, Malang, East Java, 65145, Indonesia

**Keywords:** Severe, critically ill, COVID-19, tocilizumab

## Abstract

**Background: **Several studies have revealed the potential use of tocilizumab in treating COVID-19 since no therapy has yet been approved for COVID-19 pneumonia. Tocilizumab may provide clinical benefits for cytokine release syndrome in COVID-19 patients.

**Methods: **We searched for relevant studies in PubMed, Embase, Medline, and Cochrane published from March to October 2020 to evaluate optimal use and baseline criteria for administration of tocilizumab in severe and critically ill COVID-19 patients. Research involving patients with confirmed SARS-CoV-2 infection, treated with tocilizumab and compared with the standard of care (SOC) was included in this study. We conducted a systematic review to find data about the risks and benefits of tocilizumab and outcomes from different baseline criteria for administration of tocilizumab as a treatment for severe and critically ill COVID-19 patients.

**Results: **A total of 26 studies, consisting of 23 retrospective studies, one prospective study, and two randomised controlled trials with 2112 patients enrolled in the tocilizumab group and 6160 patients in the SOC group, were included in this meta-analysis. Compared to the SOC, tocilizumab showed benefits for all-cause mortality events and a shorter time until death after first intervention but showed no difference in hospital length of stay. Upon subgroup analysis, tocilizumab showed fewer all-cause mortality events when CRP level ≥100 mg/L, P/F ratio 200-300 mmHg, and P/F ratio <200 mmHg. However, tocilizumab showed a longer length of stay when CRP <100 mg/L than the SOC.

**Conclusion: **This meta-analysis demonstrated that tocilizumab has a positive effect on all-cause mortality. It should be cautiously administrated for optimal results and tailored to the patient's eligibility criteria.

## Introduction

In December 2019, a novel virus named Severe Acute Respiratory Syndrome-Coronavirus-2 (SARS-CoV-2) that causes Coronavirus Disease-19 (COVID-19) began to spread worldwide and it become a pandemic globally ^1^. COVID-19 manifestation ranges broadly from mild symptoms to severe illness. Several studies probed multiple types of inflammatory cytokine levels and found higher levels of interleukin (IL)-1β, IL-1RA, IL-6, IL-7, IL-8, IL-10, IFN-γ, monocyte chemoattractant peptide-1, macrophage inflammatory protein (MIP)-1A, MIP-1B, granulocyte-colony stimulating factor, and tumor necrosis factor-alpha in severe COVID-19 patients ^2, 3^. COVID-19 causes severe illness due to activation of the cytokine cascade leading to cytokine release syndrome (CRS), which is delineated by systemic inflammation and multiple organ failure. Therefore, prompt strategies for treating CRS are essential for COVID-19 patients ^3– 5^.

IL-6 is a proinflammatory cytokine that plays an essential role in CRS. Activation and secretion of IL-6 by infected monocytes, macrophages, and dendritic cells cause two main effects; a plethora effect on immune cells and the innate immune system, and increased vascular permeability due to secretion of vascular endothelial growth factor (VEGF), resulting in hypotension and acute respiratory distress syndrome ^3, 5, 6^.

Tocilizumab, a humanized monoclonal antibody interleukin-6 receptor (IL-6R) inhibitor, is recommended by the National Health Commission of China for treating severe and critically ill patients with elevated IL-6 ^7^. Recently, several case reports demonstrated tocilizumab could improve the clinical manifestations of seriously ill COVID-19 patients. Several retrospective case-control, single-armed studies and randomized clinical trials declared promising results of tocilizumab treatment in SARS CoV-2 infection. Nevertheless, some systematic reviews and meta-analyses showed an unclear risk of bias and reported debatable results about tocilizumab's benefit as a treatment ^6, 8– 13^. We performed a systematic review and meta-analysis to research the risks and benefits of tocilizumab and investigate outcomes from different baseline criteria for administration of tocilizumab as a treatment for severe and critically ill COVID-19 patients.

## Methods

### Study design

We conducted a systematic review and meta-analysis to examine optimal use and baseline criteria for administration of treatment with tocilizumab versus standard of care (SOC) in severe and critically ill COVID-19 patients using data published March to October 2020. All-cause mortality events, length of stay in hospital, and days until death (time to death after first intervention) were measured to determine the risks and benefits of tocilizumab treatment. The baseline criteria for using tocilizumab included physical findings and markers of inflammation such as C-reactive protein (CRP), PaO2 and FiO2 ratio (P/F ratio), lactate dehydrogenase (LDH), D-dimer, ferritin, IL-6, leucocyte, lymphocyte count, platelet count, and procalcitonin. We performed screening of several medical databases (PubMed, Embase, Medline, and Cochrane) to collect data and calculate the risk ratio (RR) and 95% confidence intervals (95% CI). This study used similar methods for the systematic review and meta-analysis to a previous study ^14^, and was reported according to the Preferred Reporting Items for Systematic Review and Meta-Analysis (PRISMA) guidelines accessed from the PRISMA website ^15^.

### Literature search

**Figure 1.**
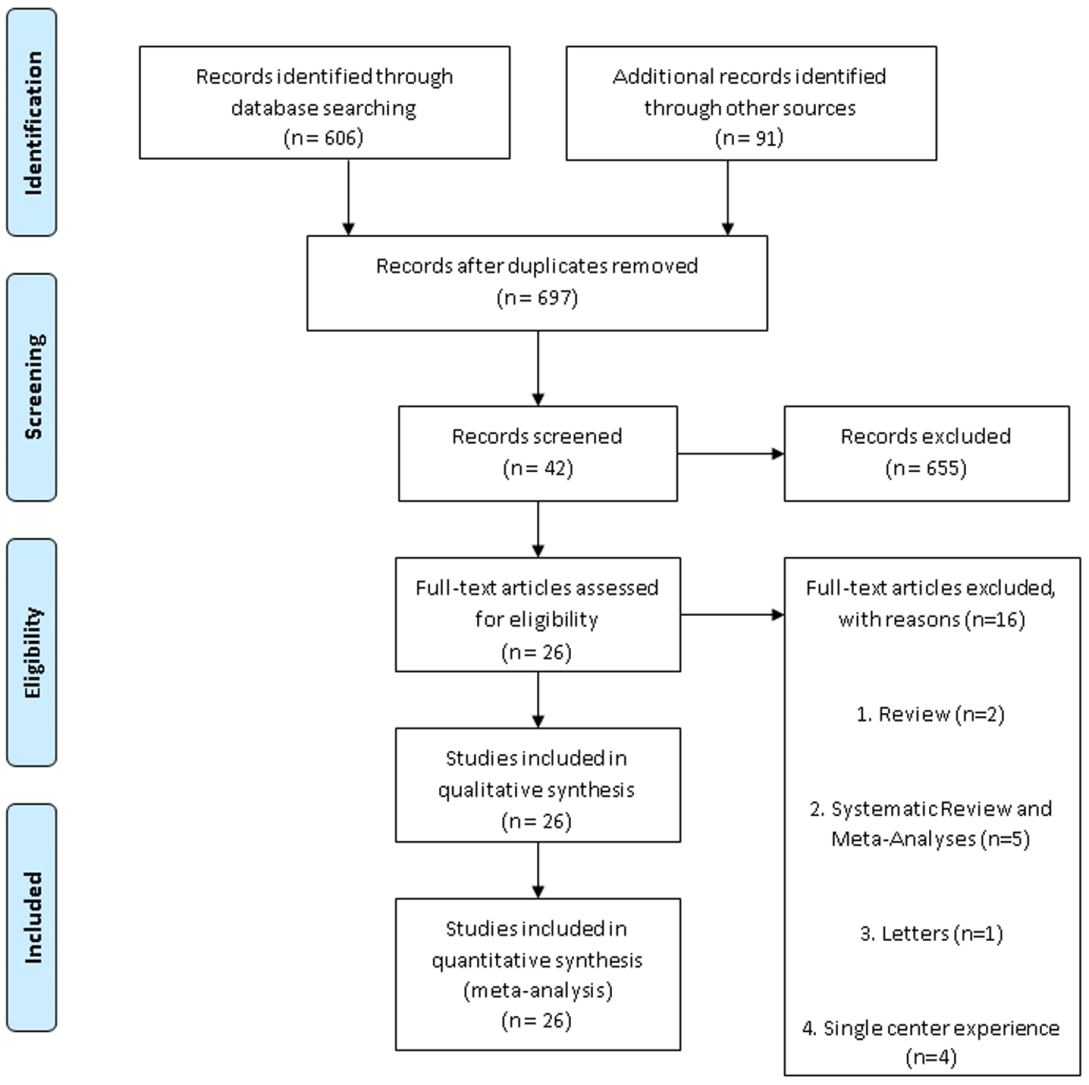
Study selection.

The search strategy ^16^, using medical subject headings (MeSH) terms, involved the use of a combination of the following keywords: (tocilizumab) OR (anti-IL-6 monoclonal antibody) OR (IL-6 blockade) OR (IL-6 receptor antagonist) AND severe AND critical ill AND (COVID-19) OR (novel coronavirus disease) OR (SARS-CoV-2). The search was performed by two authors (BAM and CW) in PubMed, Embase, Medline, and Cochrane (March 1 ^st^ to October 31 ^st^ 2020, last searched 2 ^nd^ November 2020) and the language was limited to English. We selected 606 full text and free full text articles from PubMed, included all article types, then we excluded them based on the exclusion criteria of case reports, reviews, editorials, letters, duplicate records, and studies with incomplete data. From filter selection of clinical trials, meta analyses, randomized control trials and systematic reviews within one year we got 42 articles after removing 655 articles (see Figure 1).

### Selection criteria

The studies in the three searched databases were included based on the following criteria: (1) patient confirmed for SARS-CoV-2 infection; (2) patients treated with tocilizumab and compared with the SOC; and (3) complete data were provided for clinical outcomes. Exclusion criteria were (1) case reports, reviews, editorials, and letters; (2) duplicate records; and (3) studies with incomplete data.

### Data extraction and quality assessment

All articles that qualified for inclusion according to the selection criteria were included in the analysis. Two independent investigators conducted the study assessment (BAM and CW). Two authors (BAM and EA) extracted necessary data from each included study including: first author, publication year, sample size, gender, baseline criteria for administration for tocilizumab, clinical outcomes of tocilizumab group and SOC group. Another consultant resolved any disagreement between the two investigators’ findings (ANR and TPA).

### The methodological quality assessment

We performed a methodological quality assesment of the article using the Newcastle-Ottawa Scale (NOS) before study inclusion. NOS comprises several items including: patient selection (4 points), comparability of the groups (2 points), and ascertainment of exposure (3 points). Each study was interpreted to be low quality (scores <4), moderate quality (scores of 5–6), or high quality (scores ≥7) ^17^. We only included moderate to high quality articles in the analysis. The study assessment was conducted by two independent investigators (CW and EA) using a pilot form. Another consultant resolved any disagreement between the two investigators’ findings (CWN and SDS).

### Outcomes

The study outcomes were all-cause mortality events, length of stay in hospital, and days until death (time to death after first intervention), comparing SOC and tocilizumab. We performed subgroup analysis for those outcomes based on CRP level >100 mg/L, CRP level <100 mg/L, PaO _2_:FiO _2_ ratio (P/F ratio) 200–300 mmHg, and PaO _2_:FiO _2_ (P/F ratio) <200 mmHg.

### Statistical analysis

Data were synthesized using RRs and mean differences (MDs), with 95% CIs. Significance of RRs was determined using the Z test (p<0.05 was considered statistically significant). They were assessed for heterogeneity and possibility of publication bias before calculating significancy. We used the Q test for evaluating the heterogeneity among the included studies. A random effect model was used if heterogeneity existed (p<0.10); if not, a fixed-effect model was adopted. For publication bias, we used Egger’s test and a funnel plot (p<0.05 was considered statistically significant).

We analyzed the data with Review Manager (RevMan, Cochrane, London, UK) version 5.4.1. Two authors (BAM and JKF) conducted statistical analysis and presented the results in a forest plot.

## Results

### Qualifying studies

We obtained 697 qualifying studies, 655 of which were excluded after examining the titles and abstracts. We performed a review of the complete texts for 42 potential studies and 16 studies were then excluded because they were reviews (n=2); systematic review and meta-analyses (n=5); letters (n=1); single-center experiences (n=4); case reports (n=1); brief papers (n=1) or had incomplete data (n=2). Eventually, 26 papers met the inclusion criteria for our meta-analysis; these results are summarized in Figure 1. The characteristics of studies are described in Table 1. We have summarized the results of the outcomes in Table 2.

**Table 1.** Characteristics and demographics of study.

Article	Study design	Country	Total patients	Mean/median age (years)	TCZ category **	Baseline criteria for TCZ administration	Outcome	NOS
Albertini 2020 ^18^	Single-center retrospective observational cohort study	France	44	65 (control) 64 (tocilizumab)	C	CRP ≥ 100mg/L	Clinical and laboratory marker and death	6
Biran 2020 ^19^	Retrospective multicentre observational cohort study	USA	764	65 (control) 62 (tocilizumab)	B	CRP ≥ 100 mg/L Ferritin > 900 ng/mL PaO2: FiO2 200–300 mmHg	Hospital-related mortality	8
Campochiaro 2020 ^20^	Single-center retrospective cohort study	Italy	65	60 (control) 64 (tocilizumab)	B	CRP ≥ 100 mg/L Ferritin > 900 ng/mL LDH > 220 U/L PaO2:FiO2 200-300mmHg	Safety, efficacy	8
Canziani 2020 ^21^	Retrospective case-control study	Italy	128	63 (control) 64 (tocilizumab)	A	CRP≥ 100mg/L Ferritin >900ng/mL LDH >220 U/L PaO2:FiO2 < 200 mmHg	Death	9
Capra 2020 ^22^	Retrospective observational case-control study	Italy	85	70 (control) 63 (tocilizumab)	E	PaO2:FiO2 200–300 mmHg	Survival rate	8
Colaneri 2020 ^23^	Retrospective case-control study	Italy	112	64 (control) 62 (tocilizumab)	A	CRP >50 mg/L PaO2:FiO2 200- 300mmHg	ICU admission and seven-day mortal- ity rate	8
De Rossi 2020 ^24^	Retrospective cohort study	Italy	158	71 (control) 62.9 (tocilizumab)	B or D	CRP ≥ 100mg/L LDH > 220 U/L PaO2:FiO2 200-300mmHg	Death and survival rate	7
Eimer 2020 ^25^	Retrospective cohort study	Sweden	87	58 (control) 29 (tocilizumab)	A	CRP ≥ 100 mg/Ll	30-day all-cause mortality after admission to ICU	8
Gokhale 2020 ^26^	Retrospective cohort study	India	161	55 (control) 52 (tocilizumab)	B	PaO2/FiO2 < 200mmHg	Death	8
Guaraldi 2020 ^27^	Retrospective observational cohort study	Italy	544	69 (control) 64 (tocilizumab)	A	PaO2:FiO2 200-300mmHg LDH > 220 U/L	Death or invasive mechanical ventilation	8
Gupta 2020 ^28^	Retrospective multicenter cohort study	USA	4485	63 (control) 58 (tocilizumab)	E	PaO2:FiO2 < 200 mmHg	Hazard ratios (HRs), and 30-day mortality, compared via risk differences.	8
Ip 2020 ^29^	Retrospective observational cohort study	USA	547	69 (control) 62 (tocilizumab)	B	PaO2:FiO2 200-300 mmHg	Death	8
Kewan 2020 ^30^	Retrospective cohort study	USA	51	70 (control) 62 (tocilizumab)	A	CRP ≥ 100 mg/L Ferritin > 900ng/mL PaO2:FiO2 < 200mmHg	Vasopressor support, Status hypoxia, Mortality event	7
Klopfenstein 2020 ^31^	Retrospective case-control study	France	45	71 (control) 77 (tocilizumab)	E	CRP ≥ 100mg/L PaO2:FiO2 < 200 mmHg (Ferritin and LDH were mentioned in the eligibility criteria with no exact cut-off point)	Death and/or ICU admissions	9
Masia 2020 ^32^	A prospective cohort study	Spain	138	68 (control) 62 (tocilizumab)	C	CRP < 100 mg/L Ferritin < 900ng/mL	Death, viral kinetics and antibody response	9
Mikulska 2020 ^33^	Retrospective observational single-center case-control study	Italy	196	73.5 (control) 64.5 (tocilizumab)	A	CRP > 50 mg/L Ferritin > 900ng/mL PaO2:FiO2 200- 300 mmHg	Failure-free survival	7
Moreno- Perez 2020 ^34^	Retrospective cohort study	Spain	236	57 (control) 62 (tocilizumab)	C	CRP > 50mg/L Ferritin > 1000ng/mL LDH > 300U/L PaO2:FiO2 200-300mmHg	All-cause mortality	8
Patel 2020 ^35^	Retrospective cohort study	Sweden	83	41 (control) 42 (tocilizumab)	E	CRP < 100 mg/L PaO2 : FiO2 <200 mmHg	Death	8
Potere 2020 ^36^	Retrospective case-control study	Italy	80	54 (control) 56 (tocilizumab)	D	CRP≥100mg/L PaO2:FiO2 200-300 mmHg	Requirement of invasive mechanical ventilation or death	8
Quartuccio 2020 ^37^	Retrospective single-center case-control study	Italy	111	56.2 (control) 62.4 (tocilizumab)	A	CRP < 100mg/L LDH > 220U/L	Death	9
Ramiro 2020 ^39^	Retrospective case-control single-center study	Netherland	86	67 (control) 67 (tocilizumab)	A	CRP ≥ 100mg/L Ferritin > 900ng/mL PaO2:FiO2 200-300 mmHg	Discharge from the hospital or improvement compared with baseline	9
Ramaswamy 2020 ^38^	A case-control study	USA	86	21 (control) 65 (tocilizumab)	B	CRP > 70mg/L PaO2:FiO2 200-300 mmHg	Mortality event	6
Rojas-Marte 2020 ^40^	Retrospective single-center study	USA	193	62 (control) 59 (tocilizumab)	No detail was reported	CRP ≥ 100mg/L Ferritin > 900ng/L PaO2:FiO2 200-300 mmHg	Overall mortality rate	7
Rossotti 2020 ^41^	Retrospective case-control study	Italy	222	59 (control) 59 (tocilizumab)	A	PaO2:FiO2 200-300mmHg	Overall survival analysis	8
Salvarini 2020 ^42^	Randomized controlled trial	USA	126	60 (control) 61.5 (tocilizumab)	A	CRP≥100mg/dL PaO2:FiO2 200-300 mmHg Ferritin < 900ng/L	intensive care unit with invasivemechanicalventilation, deathfromall causes, or clinical aggravation	7
Somers 2020 ^43^	Randomized controlled trial	USA	154	60 (control) 55 (tocilizumab)	A	CRP ≥ 100mg/L Ferritin > 900ng/L PaO2:FiO2 < 200mmHg LDH > 220U/L	Survival probability after intubation	6

**The dose and administration of tocilizumab are grouped into:    a. Category A: Intravenous Tocilizumab 8mg/kg bb up to 800 mg, added by a second dose after 12–24 hours    b. Category B: Single dose intravenous Tocilizumab 400mg    c. Category C: Single dose intravenous Tocilizumab 600mg    d. Category D: 324 mg of Subcutaneous injections of tocilizumab    e. Category E: not classified TCZ, tocilizumab; NOS, Newcastle-Ottawa Scale; CRP, C-reactive protein; LDH, lactate dehydrogenase; ICU, intensive care unit.

**Table 2.** Outcome and laboratory marker Tocilizumab group and standard of care group.

Outcomes	N	Mode	Value				pE	pHet	P	RR	95% CI
			SOC		TCZ						
			n	Total	n	Total					
All-cause mortality	26	Random	2475	61160	523	2112	0.2500	<0.00001	<0.00001	1.65	1.37, 2.00
Subgroup Analysis											
CRP >100 mg/L	13	Random	508	1114	234	894	0.1400	0.001	<0.0001	1.71	1.30, 2.24
CRP < 100 mg/L	7	Random	83	512	28	266	0.4500	<0.0001	0.8900	1.19	0.39, 3.59
P/F ratio 200-300 mmHg	15	Random	880	2137	296	1208	0.1300	<0.00001	0.0001	1.84	1.35, 2.50
P/F ratio <200 mmHg	8	Fixed	1576	3829	220	722	0.6600	0.8400	<0.00001	1.44	1.28, 1.63
	N	Mode	Value				pE	pHet	P	MD	95% CI
			SOC		TCZ						
Length of stay (d)	11	Random	14.22±3.21		16.81±2.25		0.5300	<0.00001	0.21	-2.05	-5.25, 1.16
Subgroup Analysis											
CRP >100 mg/L	8	Fixed	16.02±3.86		16.35±3.19		0.4800	0.7300	0.18	1.17	-0.54, 2.88
CRP < 100 mg/L	3	Fixed	10.17±1.97		18.77±1.18		0.8500	0.8700	<0.00001	-7.75	-10.31, -5.20
P/F ratio 200-300 mmHg	3	Fixed	14.28±2.73		14.28±1.53		0.8800	0.3700	0.30	1.15	-1.02, 3.31
P/F ratio < 200 mmHg	7	Random	15.36±5.73		18.74±2,23		0.7300	0.0007	0.33	-2.38	-7.19, 2.44
Days of death (d)	4	Random	13.32±3.33		6.89±6.52		0.1200	<0.00001	0.04	6.03	0.31, 11.76

Note, data were presents as mean ± SD or n [%], SOC, Standard of care; TCZ, tocilizumab; N, number of studies; CRP, C-reactive protein; RR, relative risk; MD, mean difference; pE, p Egger; PHet, p Heterogeneity; CI, confidence interval.

### Outcomes of tocilizumab treatment

**Figure 2.**
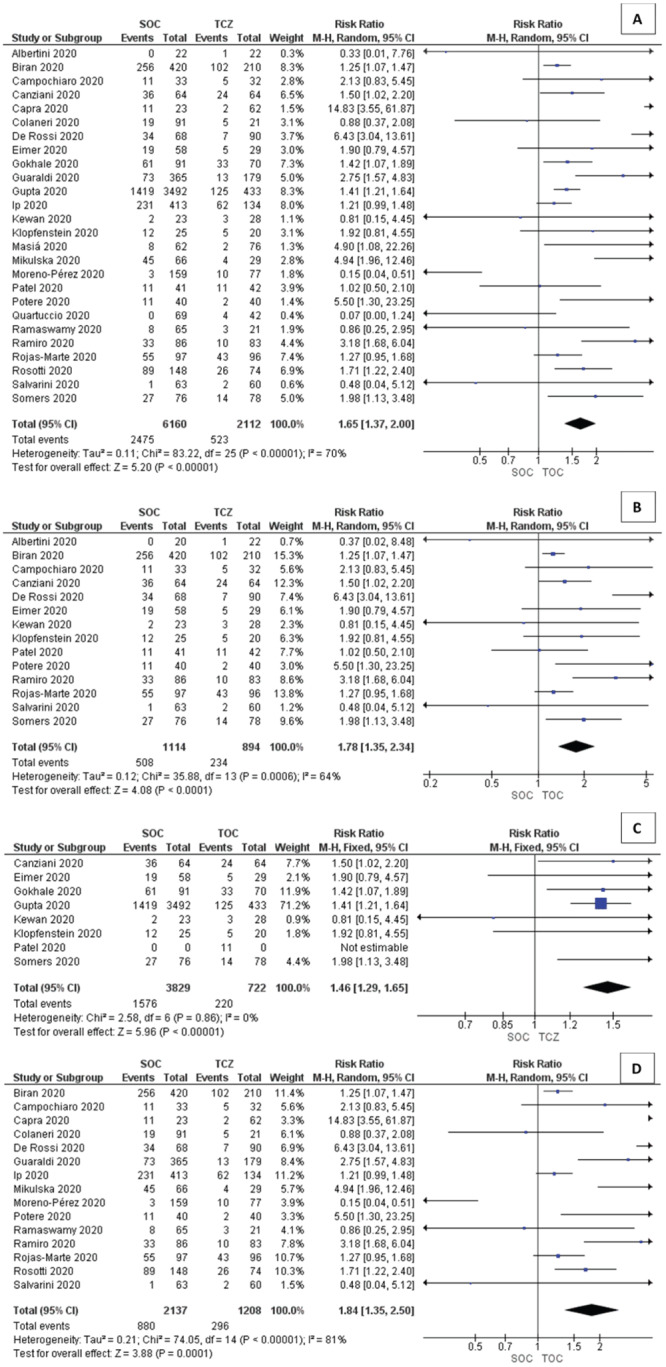
Forest plot outcome between SOC group and TCZ group. **A)** All-cause mortality event; **B)** Subgroup CRP >100 mg/L; **C)** Subgroup PaO2:FiO2 200-300 mmHg; **D)** Subgroup PaO2:FiO2 <200 mmHg. SOC, standard of care; TCZ, tocilizumab; CRP, C-reactive protein; SD, standard deviation; CI, confidence intervals.

There is a significant difference between the SOC group and tocilizumab group (RR: 1.65; 95% CI = 1.37, 2.00) from all-cause mortality events ( Figure 2) and days until death (time to death after first intervention) (MD: 6.03; 95% CI: 0.31, 11.76). There is no significant difference between the length of stay (MD: -2.05; 95% CI: -5.25, 1.16). All outcomes showed evidence of heterogeneity and the random effect model was adopted.

### Subgroup analysis

**Figure 3.**
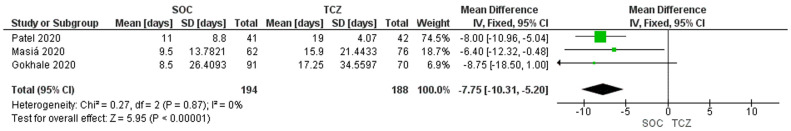
A forest plot length of stay baseline criteria for administration of tocilizumab CRP < 100 mg/L. SOC, standard of care; TCZ, tocilizumab; CRP, C-reactive protein; SD, standard deviation; CI, confidence intervals.

There is a significant difference in all-cause mortality events for patients with CRP level >100 mg/L (RR: 1.78; 95% CI: 1.35, 2.34); P/F ratio 200–300 mmHg (RR: 1.84; 95% CI: 1.35, 2.50); and P/F ratio <200 mmHg (RR: 1.44; 95% CI: 1.28, 1.63). For length of stay in hospital, CRP level <100 mg/L showed a significant difference (MD: -7.75; 95% CI: -10.31, -5.20) ( Figure 3). 

Within the subgroup analysis, evidence of homogeneity was found and we used the fixed effect model for all-cause mortality events for P/F ratio <200 mmHg and length of stay for CRP level ≥100 mg/L, CRP level <100 mg/L, and P/F ratio 200–300 mmHg. The other parameters were analyzed using the random effect model.

### Analysis of publication bias

We assessed the possibility of publication bias using Egger’s test. There was no indication of publication bias (p<0.05) for all outcomes.

## Discussion

To the best of our knowledge, this is the first meta-analysis investigating the optimal use of tocilizumab in severe and critically ill COVID-19 patients. The 26 studies analysed, mostly retrospective studies with only two clinical trials (Salvarini *et al.* and Somers *et al.*), suggest that treatment with tocilizumab gives fewer all-cause mortality events than the SOC ^18– 43^. Lan *et al.* showed that tocilizumab could not provide additional benefits for clinical outcomes of severe COVID-19, but the mortality rate was lower than the SOC, although this was not statistically different ^10^. Studies from Kaye *et al.*, Zhao, J *et al.*, and Zhao, M *et al.*, reported that tocilizumab showed a statistically significant reduction in mortality and fatality than the SOC, similar to our results ^9, 11, 13^. 

Nevertheless, hospital and ICU lengths of stay did not differ between tocilizumab and SOC ^20– 26, 31, 32, 35, 40, 43^. Only one study (Eimer *et al.*) showed that length of stay in hospital on tocilizumab was shorter than the SOC and it was able to shorten the duration of use of a ventilator. However, for the variable days until death, intervention with tocilizumab resulted in a shorter duration until death than the SOC due to secondary infections after tocilizumab treatment ^20^.

Selection criteria from included studies for using tocilizumab treatment for COVID-19 mostly included similiar clinical manifestations but baseline laboratory parameters varied. Clinical manifestations for tocilizumab treatment eligibility were frequency of respiration ≥30 breaths/min and peripheral capillary oxygen saturation (SpO2) <93% while breathing ambient air. Laboratory markers for tocilizumab treatment eligiblity were P/F ratio, CRP, ferritin, LDH and IL-6. In most studies, baseline criteria for administration of tocilizumab were level of CRP ≥100 mg/L (normal values <6 mg/L), ferritin ≥900 ng/mL (normal value <400 ng/mL), LDH >220 U/L, and P/R ratio 200–300 mmHg ^18– 20, 24, 36, 39, 40, 42^. .However, several studies used baseline criteria for administration of tocilizumab of CRP <100 mg/L and P/F ratio <200 mmHg ^23, 30– 34, 36, 43, 44^.

The SMACORE study used baseline criteria for administration of tocilizumab of CRP >50 mg/l, procalcitonin <0.5 ng/mL and P/F ratio <300 mmHg in seriously ill COVID-19 patients. Tocilizumab was first administered at 8 mg/kg (up to a maximum 800 mg per dose) intravenously, repeated after 12 hours if no side effects were reported after the first dose. The result from this study was that tocilizumab administration did not reduce mortality rate or ICU admissions ^23^.

Similar selection criteria were used by Masia *et al.*; the eligible participants had CRP >50 mg/l and tocilizumab was given at an initial dose of 600 mg intravenously for a weight of >75 kg or 400 mg when the weight was <75 kg. If their condition worsened, treatment was reevaluated following 24 hours. A second dose of tocilizumab (400 mg) was given if there was no clinical response. The result from this study was that tocilizumab administration significantly reduced the mortality rate ^32^.

In the randomized trial by Salvarini *et al.*, the selection criteria for tocilizumab treatment were P/F ratio of 200–300 mmHg. Tocilizumab was given intravenously at a starting dose of 8mg/kg until 800 mg within eight hours of randomization, and a second dose administered after 12 hours. This study showed no benefit on disease progression in the tocilizumab group compared with the SOC group ^42^.

According to the Moreno-Perez study, candidates for tocilizumab treatment had poor prognostic factors or worsening disease. One of indication for worsening condition was CRP level >100 mg/L or P/F ratio <200 mmHg ^34^.

Our subgroup analysis showed tocilizumab had a good result when CRP levels were ≥100 mg/L and P/F ratio was 200–300 mmHg or <200 mmHg. Administration of tocilizumab for CRP levels <100 mg/L did not reduce mortality and showed a longer length of stay in hospital.

There are various types of administration of tocilizumab treatment among studies. Tocilizumab can be administrated at a low dose (400 mg or 4 mg/kg) or high dose (800 mg or 8 mg/kg), as a single-dose and then continue with the second dose if clinical condition worsens in 24 hours (maximum 800 mg per dose), intravenously or subcutaneously.

## Strengths and limitations of the analysis 

Meta-analysis on this topic has not been previously conducted; only mortality events and ICU admissions have been reported by previous studies ^9– 11, 13^. In our study, we evaluate all-cause mortality events, length of stay in hospital, and days until death (time to death after first intervention) and carry out subgroup analysis of baseline criteria for administration of tocilizumab treatment. This study has a larger sample size; 2112 patients in the tocilizumab group and 6160 patients in the SOC group.

The limitations of this study are that we didn’t perform subgroup analysis outcomes according to the dosage and route of administration tocilizumab and didn’t analyze secondary outcomes after tocilizumab treatment like bacterial or fungal infections, thrombotic events, major bleeding, or requirement of invasive mechanical ventilation requirement. The results of our study should be used carefully because most studies included were retrospective and only two were randomized clinical trials, since it has been difficult to perform randomized trial during this pandemic. A meta-analysis of more clinical trial data will provide a more precise result for tocilizumab treatment in severe and critically ill COVID-19 patients.

## Conclusion

Our study provides meaningful data regarding the effect of tocilizumab in severe and critically ill confirmed COVID-19 patients. Tocilizumab is a treatment option for severe and critically ill COVID-19 patients and it appears to reduce mortality events, especially when CRP level >100 mg/L, P/F ratio 200–300 mmHg, and P/F ratio <200 mmHg. However, tocilizumab should be used cautiously according to proper selection criteria to achieve optimal results and its use should be tailored according to the eligibility of the patients. Further studies are still required, especially regarding optimal dosage and administration route of tocilizumab in COVID-19 patients.

## Data availability

### Underlying data

Figshare: Data systematic review and meta-analysis optimal use tocilizumab.zip. https://doi.org/10.6084/m9.figshare.13655894.v1 ^16^.

This project contains the following underlying data:

- TCZ_for_COVID-19.csv9

### Extended data

Figshare: Data systematic review and meta-analysis optimal use tocilizumab.zip. https://doi.org/10.6084/m9.figshare.13655894.v1 ^16^.

This project contains the following extended data:

- PubMed and Cochrane search strategies (in JPG format)

### Reporting guidelines

Figshare: PRISMA checklist for “Optimal use of tocilizumab for severe and critical COVID-19: a systematic review and meta-analysis”. https://doi.org/10.6084/m9.figshare.13655894.v1 ^16^. 

Data are available under the terms of the Creative Commons Attribution 4.0 International license (CC-BY 4.0).
