# Supplementary material for: Optimal use of tocilizumab for severe and critical COVID-19: a systematic review and meta-analysis
Source: F1000Res. 2021 Feb 4;10:73. [Version 1] doi: 10.12688/f1000research.45046.1 (PMC7953915; doi:10.12688/f1000research.45046.1)
Supplement: Peer review history

# Peer review: Reviewer response for version 1

**DOI:** 10.5256/f1000research.48118.r81031

**Published:** 2021-03-11

**Authors:** Zhongheng Zhang

**Affiliations:** 1 Department of Emergency Medicine, Sir Run Run Shaw Hospital, Zhejiang, China

This meta-analysis can provide updated evidence for an important clinical question; However, I have several comments:

1. The authors combined evidence from RCT and observational studies, which is not appropriate; the authors need to at least perform subgroup (sensitivity) analysis by restricting only high-quality trials.
2. Bayesian meta-analysis may be appropriate for down-weighing observational studies.
3. Most studies are small in sample size; the limitation of small study effect should be addressed and discussed; this effect has been well characterized in literature (Zhang *et al.*, 2013 ^1^).
4. Many observational studies may include adjusted effect size, try to combine this adjusted OR (RR) and to see whether this will change the current conclusion. 
5. Many interventional meta-analysis on COVID-19 have been published in the literature (Kim *et al.*, 2020 ^2^, Aziz *et al.*, 2021 ^3^, Zhao *et al.*, 2020 ^4^ and Kotak *et al.*, 2020 ^5^); the authors need to clarify how their study can add to the existing literature. 

Are the rationale for, and objectives of, the Systematic Review clearly stated?

Yes

Is the statistical analysis and its interpretation appropriate?

Partly

Are sufficient details of the methods and analysis provided to allow replication by others?

Yes

Are the conclusions drawn adequately supported by the results presented in the review?

Partly

Reviewer Expertise:

critical care medicine

I confirm that I have read this submission and believe that I have an appropriate level of expertise to confirm that it is of an acceptable scientific standard, however I have significant reservations, as outlined above.

## References

[1] : Small studies may overestimate the effect sizes in critical care meta-analyses: a meta-epidemiological study. *Crit Care*.2013;17(1) : 10.1186/cc11919 R2 10.1186/cc11919 23302257PMC4056100

[2] : Comparative efficacy and safety of pharmacological interventions for the treatment of COVID-19: A systematic review and network meta-analysis. *PLoS Med*.17(12) : 10.1371/journal.pmed.1003501 e1003501 10.1371/journal.pmed.1003501 33378357PMC7794037

[3] : Efficacy of tocilizumab in COVID-19: A systematic review and meta-analysis. *J Med Virol*.93(3) : 10.1002/jmv.26509 1620-1630 10.1002/jmv.26509 32918755

[4] : Efficacy of tocilizumab treatment in severely ill COVID-19 patients. *Critical Care*.2020;24(1) : 10.1186/s13054-020-03224-7 10.1186/s13054-020-03224-7 32854738PMC7450680

[5] : Use of Tocilizumab in COVID-19: A Systematic Review and Meta-Analysis of Current Evidence. *Cureus*.2020;12(10) : 10.7759/cureus.10869 e10869 10.7759/cureus.10869 33178522PMC7652362

# Peer review: Reviewer response for version 1

**DOI:** 10.5256/f1000research.48118.r79722

**Published:** 2021-03-02

**Authors:** Wiwien Heru Wiyono

**Affiliations:** 1 Department of Pulmonology and Respiratory Medicine, Universitas Indonesia, Jakarta, Indonesia

**1) Data extraction and quality assessment:**

 Two independent investigators conducted the study assessment (BAM and CW). Two authors (BAM and EA) extracted necessary data.

 I think it is not necessary to inform that one acts as an independent investigator, while he is also a member of the authors who extracted the data. Its independence is questionable.

**2) Conclusion:**

 Tocilizumab is a treatment option for severe and critically ill COVID-19 patients and it appears to reduce mortality events, especially when CRP level >100 mg/L, P/F ratio 200–300 mmHg, and P/F ratio < 200 mmHg.

1. The authors did not explain in the discussion that P/F ratio 200-300 mmHg and P/F ratio < 200 mmHg is actually different in mode.
2. When the mode of both variables is ignored, why not just conclude: when P/F ratio < 300 mm Hg.
3. Clinicians need clear conclusions, the authors' conclusions about P/F ratio 200-300 mmHg and P/F ratio < 200mm Hg are confusing.

Are the rationale for, and objectives of, the Systematic Review clearly stated?

Yes

Is the statistical analysis and its interpretation appropriate?

Yes

Are sufficient details of the methods and analysis provided to allow replication by others?

Yes

Are the conclusions drawn adequately supported by the results presented in the review?

Partly

Reviewer Expertise:

Clinical Pulmonology

I confirm that I have read this submission and believe that I have an appropriate level of expertise to confirm that it is of an acceptable scientific standard, however I have significant reservations, as outlined above.

# Response

**Published:** 2021-03-03

**Authors:** Satriyo Dwi Suryantoro

**Affiliations:** Departement of Internal Medicine, Faculty of Medicine, Airlangga University, Indonesia

Dear Prof. Wiwien,

 1) Thank you for the helpful and considerate advices.

 2) To all intents and purposes, in this research we didn't particularly examine and conclude the effect of TCZ on P/F ratio less than 300, yet we figured out significantly good impact/effect of TCZ on patients with mild ARDS (P/F ratio 200-300 mmHg), as we have mentioned on page 9 of the manuscript.

# Peer review: Reviewer response for version 1

**DOI:** 10.5256/f1000research.48118.r78921

**Published:** 2021-02-05

**Authors:** Lorenzo Cosmi

**Affiliations:** 1 Department of Experimental and Clinical Medicine, University of Florence, Florence, Italy

The manuscript “Optimal use of tocilizumab for severe and critical COVID-19: a systematic review and meta-analysis", is an exhaustive meta-analysis on studies that have evaluated the efficacy of treatment with tocilizumab in COVID-19. The meta-analysis is well performed, and the results are convincing and in line with the real life evidences, which suggest an efficacy of such a treatment in selected patients. The authors correctly underline that the treatment works in patients with high inflammatory status and with impairment of respiratory function (P/F ratio below than 300 mm Hg). The concept that tocilizumab improves the prognosis of severe COVID-19 patients, reducing hyper inflammation that contributes to vascular pulmonary damage is an important message for the scientific community.

 I have no particular concerns on this manuscript. I only suggest to quote in the introduction also a recent paper that focuses on the ability of tocilizumab to improve the lung perfusion in these patients (Salvati *et al.*, 2020 ^1^).

Are the rationale for, and objectives of, the Systematic Review clearly stated?

Yes

Is the statistical analysis and its interpretation appropriate?

Yes

Are sufficient details of the methods and analysis provided to allow replication by others?

Yes

Are the conclusions drawn adequately supported by the results presented in the review?

Yes

Reviewer Expertise:

COVID-19

I confirm that I have read this submission and believe that I have an appropriate level of expertise to confirm that it is of an acceptable scientific standard.

## References

[1] : Pulmonary vascular improvement in severe COVID-19 patients treated with tocilizumab. *Immunol Lett*.228: 10.1016/j.imlet.2020.10.009 122-128 10.1016/j.imlet.2020.10.009 33161002PMC7644186
